# Paclitaxel-eluting stents versus paclitaxel-coated balloons in coronary artery disease: a meta-analysis of randomized controlled trials

**DOI:** 10.21542/gcsp.2024.12

**Published:** 2024-03-03

**Authors:** Bryan Gervais de Liyis, Made Dhiyo Wiweka Aryaweda, Luh Oliva Saraswati Suastika

**Affiliations:** 1 Faculty of Medicine, Udayana University, Denpasar, Bali, Indonesia; 2 Department of Cardiology and Vascular Medicine, Faculty of Medicine, Udayana University, Denpasar, Bali, Indonesia

## Abstract

The efficacy of drug-coated balloons (DCB) versus drug-eluting stents (DES) for coronary artery disease (CAD) remains inconclusive. Despite paclitaxel’s common use in both DES and DCB, there is a lack of meta-analyses comparing paclitaxel-eluting stents (PES) and paclitaxel-coated balloons (PCB). This meta-analysis aimed to evaluate and compare the outcomes of DES and DCB with paclitaxel. A systematic literature search of the Medline and Cochrane databases yielded six randomized controlled trials with 951 patients (1:1 ratio). Primary endpoints were mortality, target lesion vascularization (TLV), myocardial infarction (MI), target vessel revascularization (TVR), and major adverse cardiovascular events (MACEs). Secondary endpoints included in-device binary stenosis, in-segment binary stenosis, late luminal loss (LLL), post-minimal lumen diameter (MLD), and post-diameter stenosis. Within the study populations, the incidence of previous MI was significantly lower in the PES group than in the PCB group (26.70% vs. 39.22%, OR:0.56, 95% CI [0.41–0.76], *p* = 0.0002). The meta-analysis results showed that mortality (OR:1.57, 95% CI [0.67–3.66], *p* = 0.29), TLV (OR:0.74, 95% CI [0.37–1.48], *p* = 0.39), MI (OR:1.76, 95% CI [0.79–3.88], *p* = 0.16), TVR (OR:0.76, 95% CI [0.51–1.12], *p* = 0.16), and MACEs (OR, 1.11; 95% CI [0.48–2.58]; *p* = 0.81) did not exhibit significant differences between the PES and PCB groups in CAD. Furthermore, in stent or in balloon binary stenosis (OR:0.80, 95% CI [0.34–1.87], *p* = 0.60), in segment binary stenosis (OR:1.16, 95% CI [0.48–2.80], *p* = 0.74), LLL (MD:0.03, 95% CI [−0.11 to 0.17], *p* = 0.65), post MLD (MD:0.04, 95% CI [−0.23 to 0.30], *p* = 0.77), and post diameter stenosis (MD:−5.48, 95% CI [−13.88 to 2.92], *p* = 0.20) were similar in both groups. Our comprehensive analysis concludes that both PES and PCB manifest comparable effectiveness and safety in CAD management.

## Introduction

Coronary artery disease (CAD) is a chronic disorder characterized by the accumulation of plaque in the coronary arteries, resulting in decreased blood flow to the myocardium, which can lead to angina, heart attack, or even death^1^. In 2020, approximately 20.1 million adults aged 20 years and above suffered from CAD; of these, 2 out of every 10 deaths from CAD occurred in adults under 65 years of age^2^. Minimally invasive percutaneous coronary interventions (PCI), including balloon angioplasty and stenting, are frequently used in CAD patients to enhance blood flow and reduce symptoms^3^. Plain old balloon angioplasty (POBA) is a balloon-tipped catheter used to enlarge the lumen and improve blood flow in the coronary artery at the location of stenosis or blockage^4^. This operation is relatively noninvasive and frequently accompanied by stenting^5^. Stenting, on the other hand, involves the insertion of a tiny metal mesh-like device, known as a bare metal stent (BMS), into the coronary artery to keep it open and avoid restenosis. Stenting is often performed following balloon angioplasty to offer additional support to the arterial wall^6^. BMS and POBA have been associated with several limitations, including restenosis and in-stent thrombosis, which can result in repeat revascularization procedures and decreased long-term clinical outcomes, such as major adverse cardiovascular events (MACE)^7–9^, Drug-eluting stents (DES) and drug coated balloons (DCB), on the other hand, have been shown to reduce the incidence of restenosis and improve long-term clinical outcomes, thereby reducing the need for repeat revascularization procedures^10–13^. DES and DCB function by eluting antiproliferative drugs within the coronary artery, which reduces the risk of adverse events. These drugs are designed to reduce smooth muscle cell proliferation, which is a key contributor to restenosis^14,15^.

The most commonly used drugs in DES are sirolimus, paclitaxel, and everolimus. These drugs are delivered *via* a polymer coating on the stent surface and slowly elute into the surrounding tissue over time^16–18^. Likewise, the most commonly used drugs in DCBs are paclitaxel, sirolimus, and zotarolimus^19^. The advent of DES has garnered substantial attention, with sirolimus-eluting stents (SES) and paclitaxel-eluting stents (PES) being among the most thoroughly examined and utilized varieties within this category^20^. These drugs have mechanisms of action similar to those of BMS^21^. Previous meta-analyses have compared the outcomes of DCB and DES extensively^22,23^. Sánchez et al. suggested that the use of DCB for small-vessel CAD treatment resulted in similar rates of target vessel revascularization (TVR) and restenosis compared to DES. The study also noted a lower risk of vessel thrombosis with DCB^22^. Giacoppo et al. found that using paclitaxel-coated balloon (PCB) for angioplasty was moderately less effective than repeating the procedure with a DES, in terms of reducing target lesion revascularization (TLR) after 3 years. The combination of death, myocardial infarction, or target lesion thrombosis was found to be comparable between both groups^23^. However, it is important to note that the validity of these studies is at a risk of bias in terms of comparing stents to balloons. Multiple drugs were used in each group of these studies; therefore, this heterogeneity may limit their interpretation and generalizability. These studies utilized different types of drugs in the DES group (paclitaxel, zotarolimus, sirolimus, and everolimus), which introduced significant heterogeneity. Similarly, a meta-analysis conducted by Elgendy et al. compared different drug emitting interventions, paclitaxel-coated balloons, to everolimus-eluting stents. The study showed an increased risk of TVR and TLR with PCB at extended follow-up compared to everolimus-eluting stents (EES), and better late angiographic outcomes with EES^24^. Paclitaxel is an antiproliferative agent that works by stabilizing the intracellular microtubules, preventing mitosis in the Go-G1 and G2-M phases of the cell cycle, and effectively preventing the prolonged smooth muscle cell proliferation that occurs during in-stent restenosis^25,26^. Paclitaxel is lipophilic, thus allowing rapid, homogeneously distributed cellular uptake. In addition, the lipophilic properties of paclitaxel have prolonged effects on arterial smooth muscle cells^26,27^. This property provides a promising application for the coating of balloons in the treatment of CAD^27^. Stenting also benefits from this property, as studies have shown that PES significantly reduces the need for target vessel revascularization compared to bare metal stents^28^.

Despite the growing number of studies on DES and DCB, there has been a noticeable absence of meta-analyses specifically comparing PES and PCB. The lack of comparative data makes it challenging for physicians to make informed decisions regarding the best treatment options for their patients. A comparison of PES and PCB is crucial, as paclitaxel, the drug used in both devices, has unique properties that affect performance. A meta-analysis of PES and PCB would provide a comprehensive evaluation of the safety and efficacy of these devices, allowing for the development of clinical guidelines, improved patient selection, and the optimized use of these treatments.

## Methods

### Study design and inclusion criteria

This study was conducted in accordance with the PRISMA guidelines. The meta-analysis was pre-registered and approved in the PROSPERO database (ID: CRD42023393794) before the initiation of the literature search. The inclusion criteria for this meta-analysis comprised of randomized controlled trials that evaluated the outcomes of PES and PCB in patients diagnosed with CAD. The literature search and data extraction were performed by a single author (B.G.L), and any discrepancies regarding study eligibility were resolved through consensus following review by two additional authors (M.D.W.A, L.O.S.S). The following criteria were employed: the studies had to clearly state the utilization of paclitaxel as the drug in both stents and balloons, direct comparisons of outcomes between PES and PCB were required, data of the studies had to come from registered trials, and the studies had to equally allocate participants (1:1). The use of the allocation ratio in our inclusion criteria, aimed at ensuring a balanced representation of both interventions. This methodological choice was made to minimize potential confounding variables and enhance the comparability of treatment groups, aligning with the primary objective of generating robust and clinically relevant insights. Exclusion criteria included studies that did not follow-up for at least 12 months and did not specify the type of stents or balloons utilized. This study was a retrospective study of published data and thus was exempt from IRB review.

### Literature search and selection

**Figure 1.**
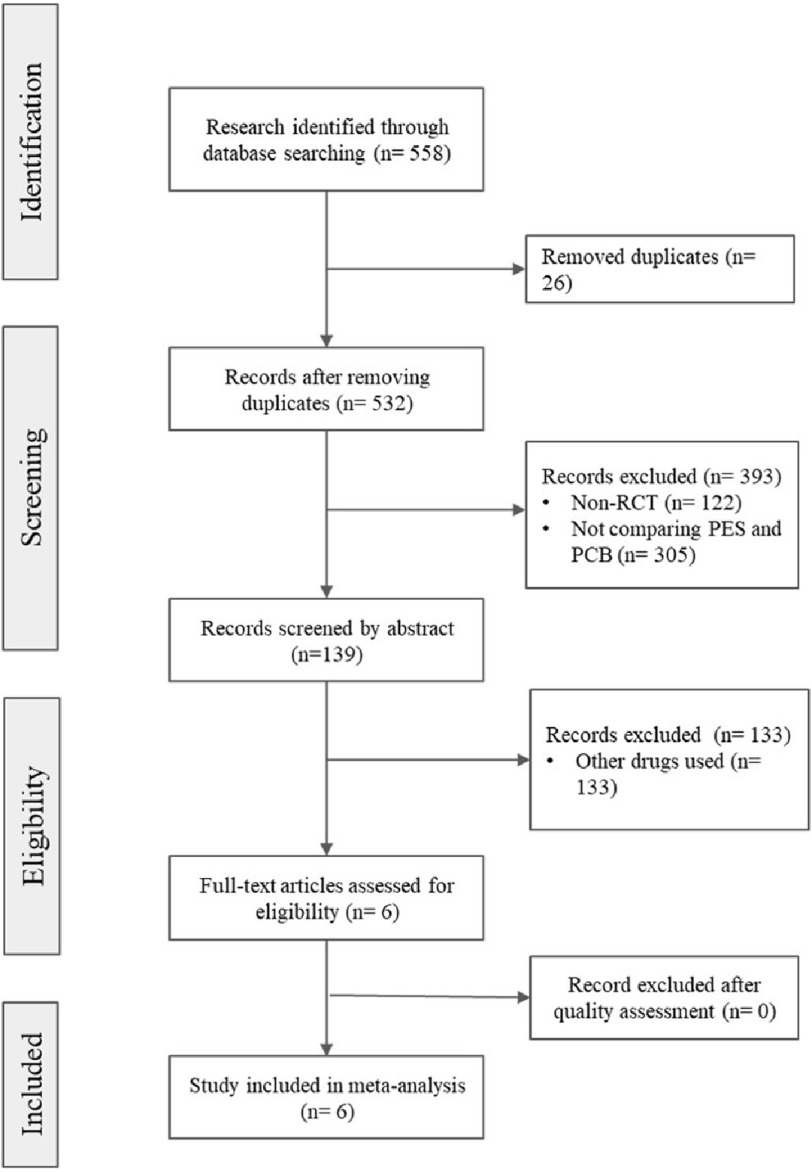
PRISMA flowchart.

A comprehensive and systematic literature search was executed in PubMed, Medline, and Cochrane databases from January 1st, 2009 to January 1st, 2023, with no language restrictions imposed. The search strategy utilized the combination of Medical Subject Headings (MeSH) terms and free-text keywords, including ((((((((((((((Coronary artery disease) OR (CAD)) OR (coronary disease)) OR (coronary heart disease)) OR (ischemic heart disease)) AND (Paclitaxel-Eluting Stents)) OR (PES)) OR (drug-eluting stents)) OR (stents)) OR (Paclitaxel Eluting Stents)) AND (Paclitaxel-Coated Balloons)) OR (PCB)) OR (drug-coated balloons)) OR (balloons)) AND (angiography). Of the 558 published abstracts or manuscripts retrieved, 132 met the pre-defined inclusion criteria, as outlined in the PRISMA flowchart (Figure 1). Further, a manual review of the references of the identified studies was performed to ensure the completeness of the search and identify any additional pertinent literature. Ultimately, six studies were selected for inclusion in the quantitative analysis.

### Quality of included studies

A systematic evaluation of the risk of bias was performed using the Cochrane Collaboration’s tool for Risk of Bias Assessment. This tool comprises seven components, which were used to assess the potential sources of bias in each study, including selection, performance, detection, attrition, reporting, and other biases. The overall quality of evidence for each outcome was subjected to a rigorous evaluation for evaluating the quality of evidence in the medical literature.

### Data extraction

The demographic, baseline clinical characteristics, and outcome variables of the included studies were systematically extracted. This information included the number of centers and participants, nation, trial registry, age, gender, presenting clinical features, history of clinical symptoms, target lesion characteristics, targeted vessel characteristics, pattern of restenosis, and type of stent or balloon utilized. The procedural details extracted encompassed parameters such as length, diameter, pressure, and inflation time. The outcome data comprised of metrics, including death, TLR, TVR, myocardial infarction (MI), MACEs, late luminal loss (LLL), post-minimal lumen diameter (MLD), post-diameter stenosis, in-stent thrombosis, in-stent binary stenosis, and in-segment stenosis.

### Statistical analysis

For binary outcomes, such as death, TLR, TVR, myocardial infarction, MACE, and in-stent binary stenosis, odds ratios (ORs) with 95% confidence intervals (CIs) were estimated. These trial-specific ORs were combined using the DerSimonian and Laird random-effects model, with the estimate of heterogeneity derived from the Mantel-Haenszel model. For continuous outcomes, such as LLL, post-MLD and post-diameter stenosis, the standardized mean difference (SMD) with 95% CIs was used as the summary statistic, and trial-specific data were pooled using the inverse variance random-effects method. The weighted mean difference (MD) and 95% CIs were calculated for continuous outcomes. An algorithm was executed to determine the mean and standard deviation, and the results were graphically depicted in Forest plots. The heterogeneity between studies was assessed using the I^2^ statistic, and the random-effects model was employed to pool effect sizes if I^2^ was greater than 50%, otherwise the fixed-effects model was utilized. All statistical analyses were performed using Review Manager software version 5.4.1 (The Nordic Cochrane Centre, The Cochrane Collaboration, Copenhagen, Denmark), and data was considered statistically significant when the *p*-value was less than 0.05.

## Results

### Study selection

The reviewed studies were selected using a flow chart established using PRISMA (Figure 1). The initial literature search conducted using PubMed, Medline, and Cochrane databases collected 558 publications. From the collected publications, 26 were identified as duplicates and removed from the study. After applying the inclusion and exclusion criteria, 6 studies were included in meta-analysis^19,20,22,23,29,30^. In this study, we analyzed several outcomes which includes death, target lesion revascularization, target vessel revascularization, myocardial infarction, late luminal loss, minimal lumen diameter, diameter stenosis, thrombosis, binary stenosis (in stent/balloon) and binary stenosis (in segment).

**Figure 2.**
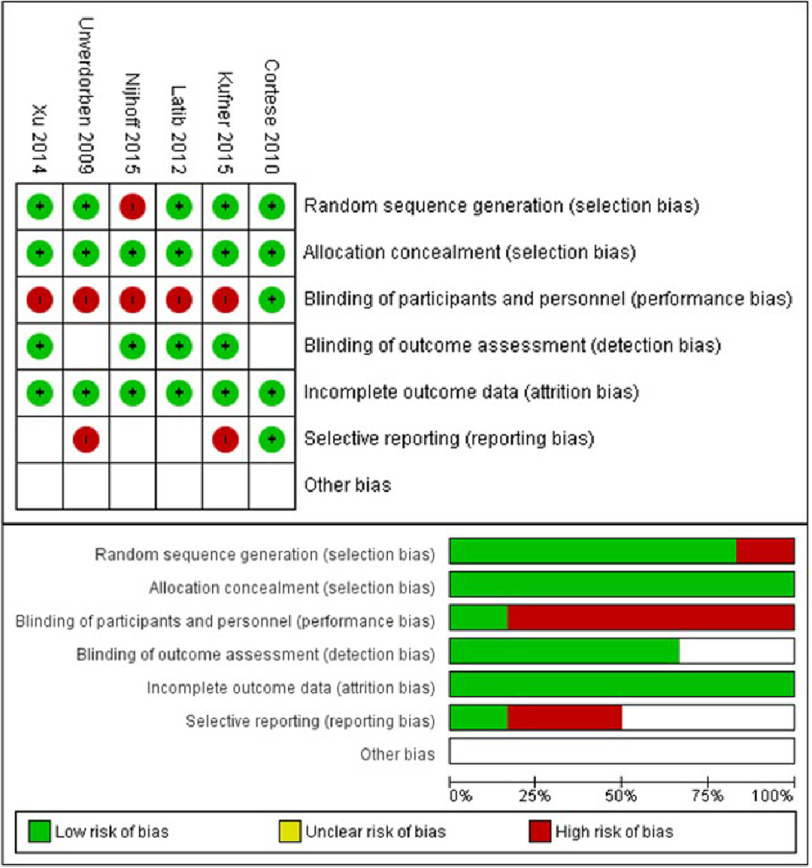
Risk of bias graph and risk of bias summary.

From the included studies a total of 951 patients were analyzed. The randomized critical trials (RCTs) in this study were assessed using RoB-2 tool by Cochrane. Out of the 6 RCTs analyzed, 5 have a high risk of bias and 1 holds concern, with five studies presenting a high risk of performance bias, one study presenting a high risk of selection bias, and 2 studies presenting a high risk of reporting bias (Figure 2).

### Characteristics of selected studies

Table 1 shows the baseline information and characteristics of the study subjects in each trial. The included studies consist of six trials from four different countries, namely Italy, Germany, Netherlands, and China. These studies were registered by the name PICCOLETO, ISAR DESIRE 3, BELLO, DEB-AMI, PEPCAD II, and PEPCAD CHINA ISR. The PICCOLETO, BELLO, and DEB-AMI trial used the radial and femoral artery as the site of access. On the other hand, the ISAR DESIRE 3 and PEPCAD CHINA ISR trial used the radial artery as the only site of access, while the PEPCAD II trial used the femoral artery as the only site of access. In total, 477 patients were treated using paclitaxel-eluting stents while 474 were treated with paclitaxel-coated balloons, in a roughly 1:1 ratio.

**Table 1:** Baseline characteristics for each study.

		Studies					
		Cortese, 2010	Kufner, 2015	Latib, 2012	Nijhoff, 2015	Unverdorben, 2009	Xu, 2014
Number of Centers		1	3	15	2	10	17
Country		Italy	Germany	Italy	Netherlands	Germany	China
Trial		PICCOLETO (EudraCTCode: 2009-012268-15)	ISAR DESIRE 3 (NCT00987324)	BELLO (NCT01086579)	DEB-AMI (NCT01086579)	PEPCAD II (NCT00393315)	PEPCAD CHINA ISR (NCT01622075)
Time		August 2008 –August 2008	August 2009 –October 2011	March 2010 –March 2012	March 2009 –June 2011	January 2006 -December 2006	March 2011 –April 2012
Access		Radial and Femoral Artery	Radial Artery	Radial and Femoral Artery	Radial and Femoral Artery	Femoral Artery	Radial Artery
Participants *S vs B* (ratio)		29 vs 28 (1:1)	131 vs 137 (1:1)	97 vs 94 (1:1)	49 vs 40 (1:1)	65 vs 66 (1:1)	106 vs 109 (1:1)
Male Participants	*S*	22	88	71	41	50	86
	*B*	28	137	94		66	109
Stent Type Used		Taxus Liberté, Boston Scientific, Natick, Massachusetts	Taxus Liberté, Boston Scientific, Natick, Massachusetts	Taxus Liberté, Boston Scientific, Natick, Massachusetts	Taxus Liberté, Boston Scientific, Natick, Massachusetts	Taxus Liberté, Boston Scientific, Natick, Massachusetts	Taxus Liberté, Boston Scientific, Natick, Massachusetts
Balloon Type Used		Dior PCB (Eurocor, Bonn, Germany)	SeQuent Please, B. Braun, Melsungen,Germany	IN.PACT Falcon paclitaxel DEB (Medtronic, Inc., Santa Rosa, California)	Second generation DIORVR coronary angioplasty balloon (Eurocor GmbH, Bonn, Germany	SeQuent Please, B. Braun, Melsungen,Germany	SeQuentPlease, B. Braun Melsungen AG, Melsungen, Germany
Mean Age (SD)	*S*	67 (10)	68.8 (10)	66.4 (9)	55.9 (9.7)	65.1 (8.7)	62.1 (9.3)
	*B*	68 (9)	67.7 (10.4)	64.8 (8.5)	57.9 (10)	64.6 (9.7)	61.8 (9.3)
Smoking	*S*	–	15	10	28	15	27
	*B*	–	19	15	21	16	23
Hyper-tension	*S*	20	101	75	15	54	69
	*B*	21	105	72	14	53	78
Diabetes	*S*	11	61	35	2	17	35
	*B*	13	56	39	5	22	44
Hyperlipidemia	*S*	13	103	73	16	46	35
	*B*	17	108	71	7	52	38
Previous MI	*S*	6	32	33	2	–	37
	*B*	5	53	46	3	–	53
Previous CABG	*S*	4	50	12		–	0
	*B*	3	15	9	0	–	3
Multi-vessels	*S*	19	122	56	-	42	22
	*B*	17	129	56	-	47	24

**Notes.**

Abbreviations B Balloon group S Stent group CABG Coronary artery bypass graft MI Myocardial infraction - No Data in Study

The average age from the stent and balloon group did not significantly differ from one another 64.22 (9.45) *vs* 64.13 (9.48) (*p* = 0.37). Patients in the stent group and the balloon group had no significant difference in gender composition, with both groups being dominated by the male gender, shown by the male percentage of 62.68% *vs* 76.16% (*p* = 0.44) respectively. The baseline characteristics were generally found to have no statistically significant difference between the stent and balloon groups (smoking [*p* = 0.82], hypertension [*p* = 0.58], diabetes mellitus [*p* = 0.26], hyperlipidemia [*p* = 0.91], previous CABG [*p* = 0.37], multivessels [*p* = 0.53]) with the exception of previous MI status in which the balloon groups were found to have a significantly higher incidence rate (26.70% *vs* 39.22%; OR 0.56 [0.41, 0.76]; *p* = 0.0002). Lesion and procedural data of each study are summarized in Table 2. Meanwhile, the demographic status and baseline characteristics are summarized in Table 3.

**Table 2:** Lesion and procedural data for each study.

		Studies											
		Cortese, 2010		Kufner, 2015		Latib, 2012		Nijhoff, 2015		Unverdorben, 2009		Xu, 2014	
		*S*	*B*	*S*	*B*	*S*	*B*	*S*	*B*	*S*	*B*	*S*	*B*
TV	Diagonal	15	15	50	59	20	26	18	18	28	20	61	47
	OMA	3	5	62	54	47	35	11	9	19	24	13	21
	PDA	11	8	56	39	28	34	20	13	17	22	34	45
													
TL	Reference Diameter	2.58 (0.24)	2.45 (0.28)	2.8 (0.49)	2.75 (0.5)	2.41 (0.4)	2.41 (0.34)	2.78 (0.53)	2.83 (0.51)	2.83 (0.36)	2.85 (0.39)	2.72 (0.44)	2.66 (0.38)
	Lesion length	11.38 (7.12)	12.41 (5.89)	10.6 (6.3)	9.6 (5.9)	14.4 (5.6)	15.4 (6.2)	16.8 (8.7)	13 (5.7)	15.4 (6.6)	15.7 (6.6)	13.08 (7.13)	12.52 (6.55)
	Lumen Diameter (pre-intervention)	0.4 (0.3)	0.48 (0.33)	0.93 (0.5)	0.97 (0.48)	0.62 (0.22)	0.6 (0.24)	0.34 (0.41)	0.29 (0.5)	0.77 (0.3)	0.74 (0.27)	0.86 (0.41)	0.85 (0.38)
	Lumen Diameter (post-intervention)	2.63 (0.23)	2.47 (0.22)	2.29 (0.44)	2.29 (0.44)	1.99 (0.28)	1.56 (0.32)	2.53 (0.41)	2.22 (0.45)	2.11 (0.78)	2.3 (0.4)	1.89 (0.75)	2.39 (0.37)
	Diameter Stenosis (Pre-intervention)	89.14 (10.6)	86.0 (12.1)	66.7 (16.5)	64.4 (16.8)	72.78 (9.27)	72.14 (10.05)	88.4 (13.6)	91.1 (15.4)	72.8 (9.4)	73.9 (8.8)	68.43 (13.25)	68.26 (12.47)
	Diameter Stenosis (Post-intervention)	9.9 (9.2)	19 (17.3)	18.5 (8.3)	18.5 (8.3)	15.42 (6.92)	29.84 (10.24)	19 (11.6)	38.4 (23.5)	11.2 (8.1)	19.5 (9.9)	27.72 (25.58)	10.51 (7.22)

**Notes.**

Abbreviations TV Target Vessel TL Target Lesion OMA Obtuse Marginal Artery PDA Patent Ductus Arteriosus

**Table 3:** Difference in basic clinical symptoms.

	PES	PCB	Odds Ratio/Mean Difference [95% CI]	*p*
Mean age	64.22 (9.45)	64.13 (9.48)	MD 0.55 [−0.65, 1.76]	0.37
Male	62.68% (299/477)	76.16% (361/474)	OR 0.68 [0.26, 1.81]	0.44
Smoking	21.21% (95/448)	21.08% (94/446)	OR 0.96 [0.69, 1.35]	0.82
Hypertension	70.02% (334/477)	72.36% (343/474)	OR 0.92 [0.69, 1.23]	0.58
Diabetes mellitus	33.75% (161/477)	37.76% (179/474)	OR 0.86 [0.65, 1.12]	0.26
Hyperlipidemia	59.96% (286/477)	60.97% (289/474)	OR 0.98 [0.74, 1.31]	0.91
Previous MI	26.70% (110/412)	39.22% (160/408)	OR 0.56 [0.41, 0.76]	0.0002
Previous CABG	18.18% (66/363)	8.15% (30/368)	OR 1.68 [0.54, 5.22]	0.37
Multivessels	60.98% (261/428)	62.90% (273/434)	OR 0.90 [0.65, 1.25]	0.53

**Notes.**

Abbreviations CABG Coronary artery bypass graft MI Myocardial infraction

### Clinical outcomes in PES- vs PCB-treated patients

**Figure 3.**
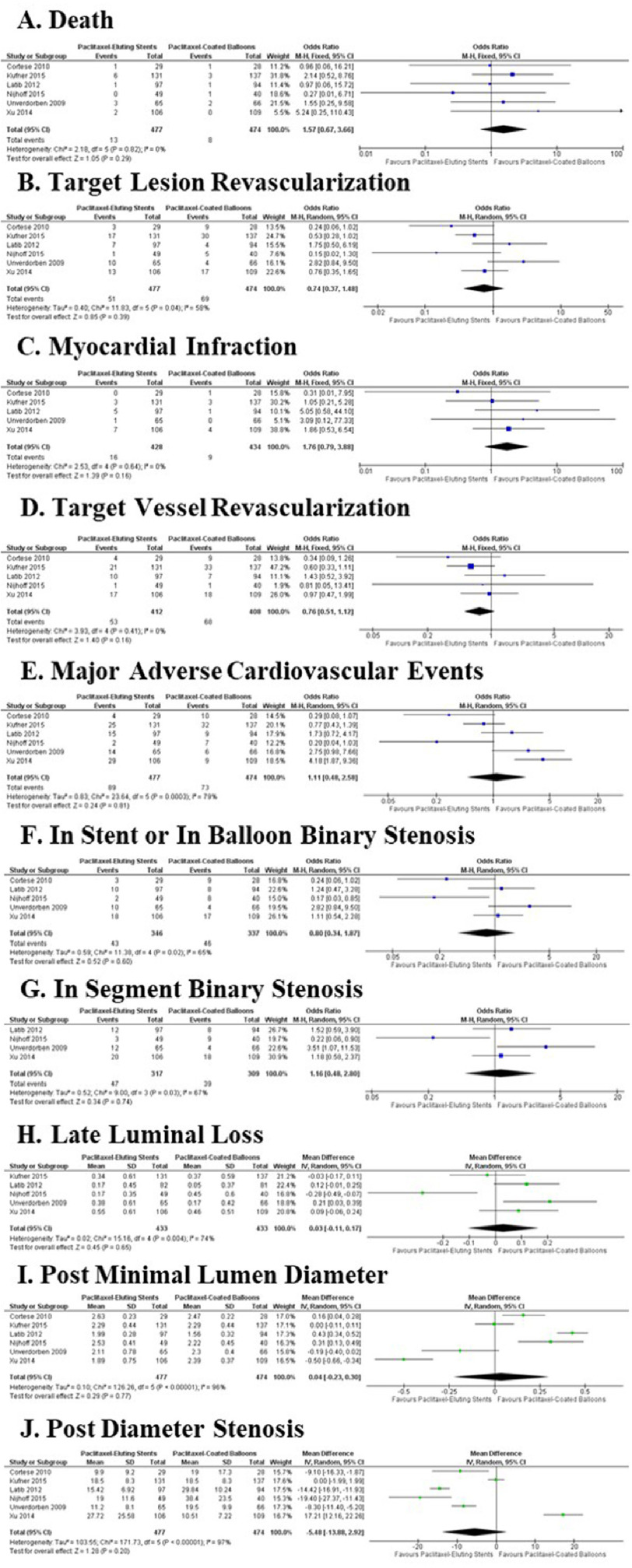
Effects of paclitaxel-eluting stents on (A) mortality, (B) target lesion revascularization, (C) myocardial infraction, (D) target vessel revascularization, (E) major adverse cardiovascular events, (F) in stent or in balloon binary stenosis, (G) in segment binary stenosis, (H) late luminal loss, (I) post minimal lumen diameter, and (J) post diameter stenosis compared with paclitaxel-coated balloons.

Clinical outcomes and complications found in the stent and balloon group and comparison between the two are summarized in Table 4. The analysis showed no significant difference in any outcome. Heterogeneity test to the death endpoint showed that there are no differences in the intervention effect across the study with an I^2^ value of 0% (*p* = 0.82) (Figure 3). The test of overall effect showed similar results, in which no significant difference between the two interventions were found (*Z* = 1.05; *p* = 0.29). All studies included resulted in a non-statistically significant odds ratio between interventions, with total odds ratio crossing the value of 1 (OR = 1.57; CI = 0.67–3.66).

The test for overall effect showed that target lesion revascularization was found to not be significantly different between the intervention groups (*Z* = 0.85; *P* = 0.39). All studies included resulted in a non-statistically significant odds ratio between interventions, with total odds ratio crossing the value of 1 (OR = 0.74; CI = 0.37–1.48). Although, different from the death outcome, there was a significant heterogeneity between the findings of each included studies (I^2^ = 58%; *p* = 0.04).

**Table 4:** Forest plot results summary.

**Endpoint**	**PES**	**PCB**	**Odds Ratio/Mean Difference [95% CI]**	***p***
Death	2.73% (13/477)	1.69% (8/474)	OR 1.57 [0.67, 3.66]	0.29
Target Lesion Revascularization	10.69% (51/477)	14.56% (69/474)	OR 0.74 [0.37, 1.48]	0.39
Myocardial Infraction	3.74% (16/428)	2.07% (9/434)	OR 1.76 [0.79, 3.88]	0.16
Target Vessel Revascularization	12.86% (53/412)	16.67% (68/408)	OR 0.76 [0.51, 1.12]	0.16
Major Adverse Cardiovascular Events	18.66% (89/477)	15.40% (73/474)	OR 1.11 [0.48, 2.58]	0.81
Binary Stenosis (in stent/in balloon)	12.43% (43/346)	13.65% (46/337)	OR 0.80 [0.34, 1.87]	0.60
Binary Stenosis (in segment)	14.83% (47/317)	12.62% (39/309)	OR 1.16 [0.48, 2.80]	0.74
Late Luminal Loss	–	–	MD 0.03 [−0.11, 0.17]	0.65
Minimal Lumen Diameter (Post)	–	–	MD 0.04 [−0.23, 0.30]	0.77
Diameter Stenosis (Post)	–	–	MD -5.48 [−13.88, 2.92]	0.20

**Notes.**

Abbreviations PES Paclitaxel Eluding Stents PCB Paclitaxel Coated Balloons

In cases of myocardial infarction, homogeneity of findings of the included studies was observed (I^2^ = 0%; *p* = 0.64). No significant difference was found between the two interventions (OR = 1.76; CI = 0.79 –3.88). The test for overall effect showed similar results (*Z* = 1.39; *p* = 0.16). The DEB-AMI^30^ trial was not included in the analysis as no data regarding myocardial infarction was presented in the published article.

In terms of target vessel revascularization, paclitaxel-eluting stents and paclitaxel-coated balloons produced statistically similar outcome (OR = 0.76; CI = 0.51, 1.12). The heterogeneity test concluded that results from the included studies were homogenic (I^2^ = 0%; *p* = 0.41). The test of overall effect concluded that no statistically significant difference can be observed between the two intervention groups (*Z* = 1.40; *p* = 0.16). The PEPCAD II^31^ trial was not included in the analysis as no data regarding target vessel revascularization was provided.

Major adverse cardiovascular events frequency was found to be statistically similar between the two intervention groups, with odds ratio confidence interval crossing the value of 1 (OR = 1.11; CI = 0.48–2.58). Significant heterogeneity was found in the results of the included studies (I^2^ = 79%, *p* = 0.0003). With the exception of the PEPCAD CHINA ISR trial^32^, all included studies showed insignificant differences between interventions. The PEPCAD CHINA ISR study found that paclitaxel-eluting stents have a higher odds of MACE than paclitaxel-coated balloons^32^. Regardless, the test of overall effect showed no significant difference between the two interventions (*Z* = 0.24; *p* = 0.81).

The odds of binary stenosis happening in stent and in balloon were found to be statistically similar between the two interventions (OR = 0.80; CI = 0.34–1.87). However, this finding was not homogenic across the included studies (I^2^ = 65%; *p* = 0.02). Unlike the other 4 included studies, the DEB-AMI trial^30^ showed that patients in the paclitaxel-eluting stents group have a lower odds of developing in stent binary stenosis compared to their counterpart (OR = 0.17; CI = 0.03–0.85). Following the trend of other measured outcomes, the test for overall effect showed no significant difference between the two interventions (*Z* = 0.52; *p* = 0.60). The ISAR DESIRE 3 trial^29^ was not included in the analysis as no data regarding the in stent and in balloon stenosis event was recorded in the published article.

In segment binary stenosis events, frequency does not differ significantly between the interventions (OR = 1.16; CI = 0.48–2.80). Heterogeneity test results showed that not all included studies have the same conclusions (I^2^ = 67%; *p* = 0.03). The PEPCAD II^31^ concluded that patients treated with paclitaxel-eluding stents have a higher odds of developing in-segment binary stenosis compared to their counterparts. Nevertheless, the test for overall effect concluded that no significant difference is present between the two interventions regarding the in segment binary stenosis events (*Z* = 0.34; *p* = 0.74). The ISAR DESIRE 3 trial^29^ and the PICCOLETO trial^33^ were not included in the analysis as the published articles lacks data regarding in segment binary stenosis event.

Late luminal loss amount does not differ significantly between interventions as analysis showed that the mean difference’s confidence interval crosses zero (MD = 0.03 mm; CI = −0.11–0.17 mm). This finding is not homogenous, as shown by the heterogeneity test (I^2^ = 74%; *p* = 0.004). The PEPCAD II trial^31^ mentioned a mean difference of 0.21 mm with the confidence interval ranging from 0.03 to 0.39 mm, making it the only included study which confidently concludes that difference in late luminal loss is significantly present between the intervention. Nonetheless, the test for overall effect showed no significant difference between the two groups (*Z* = 0.45; *p* = 0.65). The PICCOLETO trial^33^ was not included in this analysis as the published article lacked data regarding late luminal loss.

The post-intervention minimal lumen diameters are not significantly different between the two interventions with confidence interval of the mean difference crossing zero (MD = 0.04 mm; CI = −0.23–0.30 mm). The findings from the 6 included studies are very heterogenic (I^2^ = 96%; *p* < 0.00001), with 2 studies^29,31^ showing no significant difference between the interventions, 3 studies^30,33,34^ showing wider diameter in favor of the paclitaxel-eluting stents group, and a study^32^ showing wider diameter in favor of the paclitaxel-coated balloons group. Nevertheless, the test for overall effect concluded that the two intervention groups does not differ significantly (*Z* = 0.29; *p* = 0.77).

Post-intervention percent diameter stenosis was found to be statistically similar when calculated in total (MD = −5.48%; CI = −13.88–2.92). Similar to post-intervention luminal loss, high heterogeneity was found regarding the percent diameter stenosis outcome of each study (I^2^ = 97%; *p* < 0.00001), in which 4 studies^30,31,33,34^ concluded that patients treated with paclitaxel-eluting stents have a lower post-intervention percent diameter stenosis, one study found no significant difference between the two groups^29^, and one study^32^ found that patients treated paclitaxel-coated balloons have a lower post intervention percent diameter stenosis. Following the trend of the other clinical outcomes, test for overall effect found no significant difference between the two interventions (*Z* = 1.28; *p* = 0.20).

## Discussion

Careful consideration of the type and location of the stenosis or occlusion, as well as the overall health and medical history of the patient, is essential in determining the best treatment strategy for PCI. Our meta-analysis found homogenic, non-significant difference in the outcome of death, target vessel revascularization, and myocardial infarction events. This indicated that PES and PCB are equally effective at preventing said events in patients with coronary artery disease. This finding is consistent with the findings of a previous meta-analysis conducted by Li et al. ^26^, which concluded that drug-coated balloon is noninferior to drug-eluting stent in terms of delivering a good outcome in nonfatal myocardial infarction in patients with de novo small coronary artery vessel disease. Similar results were found in the meta-analysis performed by Elgendy et al. ^24^*,* which concluded that PCB was associated with similar risk of target vessel revascularization at 1-year follow up in patients with ISR.

On the other hand, highly heterogeneous results were found in the outcomes regarding TLR, MACE, binary stenosis (both in stent/balloon and in segment), late luminal loss, and minimal lumen diameter and diameter stenosis, both of which reached *p* values of <0.00001 and I^2^ of 96% and 97%, respectively. The high heterogeneity might have been caused by the difference in the race composition of the patients included in each included study in this meta-analysis. Different races, such as white, black, Hispanic, and Asian, have been shown to have significantly different outcomes with regard to major adverse cardiac event rates, even after adjusting for differences in baseline characteristics^35^. Thus, it is advisable to conduct additional research to explore the potential role of race in determining the outcomes of both PES and PCB.

Another important factor that should be considered is the different types of balloons used in each included study. The ISAR DESIRE 3^29^, PEPCAD II^31^, and PEPCAD CHINA^32^ trials used the SeQuent^®^ Please catheter, the PICCOLETO trial^33^ used the DIOR^®^ Paclitaxel-eluting coronary balloon catheter, the DEB-AMI trial^30^ used the second-generation DIOR Paclitaxel-eluting coronary balloon catheter, and the BELLO trial^34^ used the IN. PACT™ Admiral™ Paclitaxel-coated PTA Balloon Catheter.

DIOR and Sequent catheters use different coating technologies. The former achieves paclitaxel delivery by coating it on a roughened surface of the balloon, whereas the latter achieves paclitaxel delivery by sticking paclitaxel into a water-soluble matrix.

The SeQuent^®^ Please technology allows for higher bioavailability as the drug is released completely after the first expansion^33^. We found that trials using the SeQuent^®^ Please catheter resulted in an odds ratio in favor of PCB in the MI, stent or balloon binary stenosis, segment binary stenosis, and mortality outcome, while resulting in favor of PES in the post-intervention minimum lumen diameter, late luminal loss, and target vessel revascularization.

However, it is important to note that most of these results are not statistically conclusive since the confidence interval crosses the value of one. In addition, we found that the BELLO study^34^, the only included study using the IN.PACT™ Admiral™ Paclitaxel-coated PTA Balloon Catheter, which uses urea as the inactive ingredient to facilitate the release and transfer of paclitaxel, was the only study which resulted in favor of PCB in the TVR outcome, although the result is not statistically conclusive as the confidence interval still crossed the value of one.

This trial also favored PCB in 8 other outcomes, namely MI, TVR, TVL, MACE, stent or balloon binary stenosis, segment binary stenosis, post-intervention late luminal loss, and minimal lumen diameter, with the last outcome being the only significantly conclusive result.

Several limitations must be acknowledged when interpreting the results of our meta-analysis. First, the included trials varied in design, patient population, and follow-up duration, potentially introducing heterogeneity. Additionally, the relatively small sample size of the selected studies may limit the generalizability of our findings. Furthermore, the paucity of long-term data on outcomes such as late stent thrombosis or restenosis rates necessitates the cautious interpretation of our conclusions beyond the observed follow-up periods. Moreover, our study included trials with various catheters used for PCB, which allowed for bias based on the individual performance of each catheter. In addition, high heterogeneity in some of the observed outcomes may require clinicians to approach the conclusions of this meta-analysis in the general population more carefully. Despite these limitations, our meta-analysis provides valuable insights into current evidence comparing PES and PCB in the context of CAD.

## Conclusions

Our meta-analysis aimed to compare the clinical outcomes of PES and PCB use in patients with CAD. We analyzed death, target vessel revascularization, target lesion revascularization, MACE, myocardial infarction, in stent/balloon binary stenosis, in segment binary stenosis, late luminal loss, minimal lumen diameter, and diameter stenosis outcomes. We found no significant difference between the two interventions in all outcomes, despite the highly heterogeneous results. This finding implies that PCB and PES are similarly effective in terms of efficacy and safety in the treatment of CAD.

## Conflict of Interest

The authors declare no potential conflicts of interest with respect to the research, authorship, or publication of this article.

## Acknowledgements

No other acknowledgements

## Sources of Funding

This research did not receive any specific grant from funding agencies in the public, commercial, or not-for-profit sectors

## Author Contributions

Conceptualization: Bryan Gervais de Liyis

Supervision: Bryan Gervais de Liyis and Luh Oliva Saraswati Suastika

Software: Bryan Gervais de Liyis

Writing -Original Draft Preparation: Made Dhiyo Wiweka Aryaweda

Writing -Review & Editing: Made Dhiyo Wiweka Aryaweda, and Luh Oliva Saraswati Suastika
